# Exploring Burnout at the Morgue During the COVID-19 Pandemic: A Three-Phase Analysis of Forensic and Pathology Personnel

**DOI:** 10.3390/healthcare13050504

**Published:** 2025-02-26

**Authors:** Lilioara-Alexandra Oprinca-Muja, Adrian-Nicolae Cristian, Elena Topîrcean, Alina Cristian, Marius Florentin Popa, Roxana Cardoș, George-Călin Oprinca, Diter Atasie, Cosmin Mihalache, Mihaela Dana Bucuță, Silviu Morar

**Affiliations:** 1 Faculty of Medicine, Lucian Blaga University of Sibiu, 550169 Sibiu, Romania; lilioaraalexandra.muja@ulbsibiu.ro (L.-A.O.-M.); elena.topircean@ulbsibiu.ro (E.T.); alina.cristian@ulbsibiu.ro (A.C.); georgecalin.oprinca@ulbsibiu.ro (G.-C.O.); atasie.diter@ulbsibiu.ro (D.A.); cosmin.mihalache@ulbsibiu.ro (C.M.); mihaela.bucuta@ulbsibiu.ro (M.D.B.); silviu.morar@ulbsibiu.ro (S.M.); 2 Faculty of Medicine, Ovidius University of Constanța, 900527 Constanța, Romania; marius_popa2005@yahoo.com; 3 Department of Clinical Psychology, Babeș-Bolyai University of Cluj Napoca, 400347 Cluj-Napoca, Romania; roxana.cardos@ubbcluj.ro

**Keywords:** burnout, forensic medicine, pathology, COVID-19, pandemic

## Abstract

**Background/Objectives**: Burnout is a critical concern among healthcare professionals, particularly during crises such as the COVID-19 pandemic. This study investigated burnout levels among forensic medicine and pathology personnel at three distinct phases: the early pandemic period (Phase 1—September 2020), the peak of the pandemic (Phase 2—October 2021), and the post-pandemic period (Phase 3—October 2024). **Methods**: A total of 37 participants employed in forensic medicine and pathology departments completed the Maslach Burnout Inventory (MBI). A one-way repeated measures ANOVA was conducted to assess within-subject differences over time. Normality and sphericity were tested using the Shapiro–Wilk test and Mauchly’s test, with the Greenhouse-Geisser correction. Post hoc Bonferroni-adjusted comparisons identified significant differences, and partial eta squared (η^2^) was reported for effect sizes. **Results**: Results showed significant fluctuations in burnout levels across the three phases. Emotional exhaustion and low personal accomplishment peaked during Phase 2, with slight reductions observed in Phase 3. Gender differences were evident, with females reporting higher EE levels and males exhibiting higher depersonalization across all phases. Marital and parental status also influenced burnout levels, with unmarried individuals and those without children showing higher burnout scores. Medical doctors experienced the highest burnout levels among professional roles, while auxiliary staff showed significant challenges in the PA subscale. **Conclusions**: The COVID-19 pandemic was pivotal in exacerbating burnout levels due to increased workload, crisis decision-making, and emotional toll. Although the sample size is limited, these findings underscore the importance of implementing targeted interventions to mitigate burnout among forensic and pathology personnel, especially during healthcare emergencies. Gender-based differences in burnout suggest the necessity of specific **workplace well-being strategies**, while the protective role of family status demonstrates the importance of **work-life balance policies**. The persistence of psychological distress after a medical crisis calls for **long-term monitoring and support programs.** There is a need for improved workload distribution, peer support networks, and mental health training to build resilience among forensic and pathology personnel.

## 1. Introduction

The concept of burnout as a psychological phenomenon secondary to work-related stress emerged in the 1970s with Freudenberger’s work [1]. By the 1980s, Christina Maslach introduced the term while interviewing human service workers to understand how they managed stressful situations [2]. Together with her colleagues, Maslach developed a measurement scale during their study, which laid the foundation for the widely recognized Maslach Burnout Inventory (MBI) used today for diagnosing clinical burnout [3]. The three pillars of burnout are emotional exhaustion, depersonalization, and low personal accomplishment. Emotional exhaustion refers to feelings of being emotionally drained after prolonged interaction with others and is considered the core dimension of burnout [4]. Depersonalization results from emotional exhaustion and manifests as negative attitudes or excessively detached responses toward other people or clients [4]. Finally, reduced personal accomplishment reflects a diminished sense of achievement or success at work [4]. Regarding risk factors for burnout syndrome, most studies identify three main groups that contribute to chronic stress and, consequently, emotional exhaustion, depersonalization, and reduced personal accomplishment. These factors are socioeconomic, occupational, and psychological in origin [5].

From a clinical perspective, burnout syndrome is not yet considered a psychiatric disorder, as it is not included in the Diagnostic and Statistical Manual of Mental Disorders (DSM-5-TR, 2022). However, burnout is classified as a “state of vital exhaustion” under “problems related to life-management difficulty” in the World Health Organization’s International Classification of Diseases (ICD-10, 2010) [6]. The latest ICD-11 defines burnout as “a syndrome conceptualized as resulting from chronic workplace stress that has not been successfully managed”, classified as an occupational phenomenon, not a medical condition [7]. To assess and diagnose clinical burnout, several scales have been developed over the years. The most widely recognized and used scale is the Maslach Burnout Inventory (MBI). Other commonly used scales include the Burnout Measure (BM), the Copenhagen Burnout Inventory (CBI), the Oldenburg Burnout Inventory (OLBI), the Shirom-Melamed Burnout Questionnaire (SMBQ), and its later version, the Shirom-Melamed Burnout Measure (SMBM) [8]. Clinicians often associate burnout syndrome with severe stress, adjustment disorder, undifferentiated somatoform disorder, or major depression; however, it appears that “work-related neurasthenia” most closely aligns with the clinical manifestations of burnout [6].

Forensic medicine workers (FMW) are exposed to various stressors due to the nature of their work, which includes evaluating traumatic events, handling deceased individuals, and interacting with victims’ families [9]. Burnout and occupational stress are prevalent among these individuals due to the technical, emotional, and organizational demands of their roles [10]. High levels of emotional exhaustion, post-traumatic stress syndrome (PTSD), and secondary traumatic stress (STS) are prevalent in this group [11], exacerbated by prolonged exposure to graphic scenes and high emotional cases such as infant deaths or mass fatality incidents but also organizational challenges, such as inadequate managerial support, high caseloads, and role conflicts [9]. Subgroups such as autopsy technicians and forensic physicians are particularly vulnerable, experiencing significant emotional exhaustion and PTSD symptoms, especially after exposure to critical events involving children [10]. Women and professionals with extensive work hours or teaching responsibilities are also exposed to a higher risk of burnout [9]. The COVID-19 pandemic further exacerbated these challenges, intensifying stress levels through heightened infection risks and increased workloads, thereby contributing to higher emotional and psychological strain in this already high-stress profession [10].

Regarding pathologists, burnout is increasingly recognized as a significant concern, driven by factors such as heavy workloads, administrative complexity, and emotional exhaustion [12]. Common manifestations include high levels of stress, depersonalization, and reduced personal achievement, exacerbated by demands for productivity and under-recognition of their contributions, but also by understaffing, lack of compensation for extra work, and complex reporting requirements [12]. Studies indicate burnout rates in pathologists range from 33% to 45%, with medical laboratory technologists showing even higher rates (up to 73%) [12].

In December 2019, the World Health Organization (WHO) reported cases of pneumonia of unknown etiology in Wuhan, China. As the number of cases increased, research into the cause began, and on 7 January 2020, a new type of coronavirus, later named SARS-CoV-2, was isolated. With the rapid global spread of the virus and the emergence of numerous cases, the WHO declared the coronavirus outbreak a pandemic on 11 March 2020. During this pandemic, scientists worldwide focused on developing effective treatment plans and vaccines while also studying the specific pathophysiology of the novel coronavirus through clinical cases, microscopic specimens, and therapeutic approaches. However, relatively few efforts have been made to explore the impact of the SARS-CoV-2 pandemic on healthcare workers, who bore the brunt of the crisis on the frontlines, confronting the aggressive and unpredictable nature of this newly discovered virus.

The COVID-19 pandemic has significantly impacted mental health, causing widespread emotional distress due to stressors like uncertain prognoses, resource shortages, financial losses, and restrictive public health measures [13]. Common reactions include anxiety, depression, insomnia, and increased substance use, with vulnerable groups such as infected individuals, those with preexisting conditions, and healthcare providers being at a heightened risk [13]. In residential care homes, the rapid spread of COVID-19 has overwhelmed healthcare workers, leading to excessive workload and high emotional exhaustion [14]. Quarantine measures have intensified these effects, leading to frustration, stigma, and financial strain [13]. Also, the COVID-19 pandemic significantly altered working conditions, contributing to heightened occupational stress, and was associated with physiological alterations like endocrine system disorders [15]. Healthcare workers faced unprecedented mental health challenges during the COVID-19 pandemic, with stressors including fears of contagion, extended workloads, changing protocols, and the emotional toll of patient care and mortality [16]. Common psychological reactions included acute stress, anxiety, depression, PTSD, and moral injury [16]. Alcohol consumption, a widespread maladaptive coping mechanism in Romania [17], further exacerbated these reactions. ICU and emergency units worked tirelessly to keep patients alive, but for those with adverse outcomes, their final destination was the morgue. Here, medical personnel delivered the bodies to the next of kin in two body bags, sealed within a coffin. Autopsies were conducted only in forensic cases, for scientific purposes, or to confirm SARS-CoV-2 infection in patients without rt-PCR results. The risk of infection during these autopsies was significant, contributing to exceptionally high stress levels among the staff involved. The COVID-19 pandemic significantly altered the work environment for forensic medicine and pathology personnel, imposing new challenges and stressors that contributed to burnout. Forensic and pathology professionals were required to rapidly adapt to evolving safety protocols, including the implementation of stringent infection control measures such as personal protective equipment use, working in specialized autopsy rooms and handling post-mortem samples with increased precautionary measures. Additionally, forensic and pathology teams faced an increased workload due to the high number of COVID-19-related deaths, further exacerbating job-related stress. The pandemic also intensified medico-legal responsibilities, as forensic experts played a critical role in clarifying causes of death, addressing concerns regarding medical malpractice, and managing legal disputes related to hospital-acquired infections [18,19].

## 2. Materials and Methods

### 2.1. Participants

The study included 37 participants from the Sibiu County Morgue, Romania, comprising personnel from the forensic medicine department and the pathology department. The decision to conduct a single-center study was made to ensure a homogeneous sample while maintaining a high participation rate, avoiding self-selection bias, even if this resulted in a smaller study population. Among these participants, 20 were males and 17 were females, aged 25 to 59 years. Most participants were in the 50–59 age group (N = 14), while the 20–29 (N = 5) and 30–39 (N = 5) age groups were the least represented. Fourteen participants were medical doctors, including nine primary physicians and five medical residents. The remaining participants included eleven medical assistants, four autopsy practitioners, two drivers, three medical registrars, and three cleaning attendants. Most of the participants included in the study were married (N = 27), four individuals were in a relationship, five staff members were divorced, and only one was a widower. Twenty-four participants had children in their care at the time of the study. Most participants resided in urban areas (N = 34), while 3 were from rural areas. (Table 1).

### 2.2. Eligibility Criteria

For participant selection, we drafted several inclusion and exclusion criteria. The inclusion criteria were active employment in forensic medicine or pathology during the study period, a minimum of six months of professional experience, and completing and submitting the full questionnaire with no missing responses. The exclusion criteria included individuals on extended leave, for example, medical leave, maternity leave, or sabbaticals, during the study period, professionals working part-time with limited case exposure, and incomplete questionnaire responses.

### 2.3. Assessment Tools

For the assessment of burnout syndrome, we used the original Maslach Burnout Inventory with 25 items, validated by Christina Maslach and Susan E. Jackson [2]. The questionnaire consists of 25 items and is structured across three dimensions: Emotional Exhaustion (9 items), Depersonalization (6 items), and Reduced Personal Accomplishment (10 items). Responses were measured using a 5-point Likert scale: 1—very rarely, 2—rarely, 3—sometimes, 4—frequently, and 5—very frequently often. The advantage of this scale lies in its ability to provide a greater variety of responses, thereby reducing the risk of receiving uniform answers from most participants. To counteract the potential monotony of responses, reverse-scored items were included within the questionnaire, requiring participants to pay closer attention to their wording and encouraging consistency in their answers. The Cronbach’s alpha coefficient for the scale in our sample was 0.752, indicating acceptable internal consistency. The total scores for each subscale are categorized into low, medium, or high burnout levels based on predefined thresholds. The total score for each subscale is calculated by summing the responses to the respective items. Burnout severity is then categorized into low, moderate, or high levels based on predefined cutoff values. (Table 2).

### 2.4. Study Design

The present study analyzed burnout levels among personnel from the pathology and forensic departments at three distinct points in time (phases), chosen to reflect the impact of the COVID-19 pandemic on workplace stress. Data collection occurred as follows:

- Phase 1 (September 2020): This period marked the beginning of the second wave of the COVID-19 pandemic in Romania, shortly after the national state of emergency ended and transitioned into a state of alert. By this time, the first wave of infections (starting in March 2020) had subsided, with daily deaths averaging around 40 nationwide. The primary challenge for healthcare personnel during this phase was the lack of scientific knowledge about the virus, including its behavior and aggressiveness, which caused significant uncertainty. Research efforts were beginning, and the stressful workload was manageable compared to later phases;
- Phase 2 (October 2021): This phase corresponded to the peak of the fourth wave of infections, driven by the extremely aggressive Delta variant. During this wave, Romania experienced approximately 600 deaths per day at the national level, with 10–15 deaths per day in Sibiu County. By this time, the medical staff had already endured two additional waves (Beta and Delta variants) during the winter of 2020 and spring of 2021, each resulting in around 200 daily deaths nationwide. The fourth wave introduced unprecedented challenges, including severe shortages of protective equipment, overcrowding in morgues, the use of improvised refrigerated containers for deceased bodies, and the need for extensive autopsies. Personnel faced immense physical and emotional strain, further exacerbated by a lack of resources such as stretchers and adequate storage facilities for the deceased;
- Phase 3 (October 2024): By this period, the SARS-CoV-2 virus had ceased to pose a significant public health threat. Following the dominance of the less aggressive Omicron variant and widespread vaccination coverage, COVID-19-related deaths had decreased to near zero. Unique protocols for handling and managing such cases were no longer in effect. Since the summer of 2022, normal operations have continued in the pathology and forensic departments.

Data from these three points in time were analyzed to evaluate the progression and variation of burnout levels in relation to the distinct phases of the pandemic. This approach allowed the identification of key stressors and their impact on staff psychological well-being over time.

To enhance clarity and present well-structured results, we formulated several research questions based on the collected data. These questions guided the development of our study hypotheses:

- Did the COVID-19 pandemic influence burnout levels among individuals working in forensic medicine and pathology, particularly in morgue settings?
- Were there significant differences in emotional exhaustion, depersonalization, and personal accomplishment across the different phases of the pandemic?
- Did the COVID-19 pandemic have a differential impact on burnout levels based on gender, age, and occupational role within forensic and pathology personnel?
- To what extent did marital status and parenthood influence burnout levels in crisis situations such as the COVID-19 pandemic?
- Can burnout levels decrease following the resolution of a prolonged crisis?

Based on preliminary observations and the formulated research questions, we developed several hypotheses aligned with our three-phase study model:

- H1: Total burnout scores increase significantly during the peak of the COVID-19 pandemic among individuals working in the morgue;
- H2: High emotional exhaustion and depersonalization, along with low personal accomplishment, are more pronounced in forensic and pathology personnel during the COVID-19 pandemic;
- H3: Female professionals tend to exhibit higher levels of burnout during crisis periods such as the COVID-19 pandemic, with emotional exhaustion being the most predominant dimension;
- H4: Medical doctors experience the highest levels of burnout among forensic and pathology personnel during the COVID-19 pandemic;
- H5: Being married or having children may serve as a protective factor against burnout during crisis periods;
- H6: Burnout levels may decrease after the end of a crisis period, such as the COVID-19 pandemic, but they are unlikely to return to pre-crisis baseline levels.

### 2.5. Data Analysis

A one-way repeated measures ANOVA was conducted to assess the differences in Maslach Burnout Inventory scores across three time points: September 2020 (Time 1), October 2021 (Time 2), and October 2024 (Time 3). The analysis focused on four subscales of the Maslach Burnout Inventory: Emotional Exhaustion, Depersonalization, Personal Achievement, and the overall Burnout total score. This statistical test was chosen to evaluate within-subject changes over time for each variable, assessing whether the mean scores differed significantly across the three measurement points. Before the analysis, the data were inspected for normality and sphericity assumptions: normality was assessed using the Shapiro–Wilk test and visual inspection of Q–Q plots, and sphericity was evaluated using Mauchly’s test. In cases where the assumption of sphericity was violated, the Greenhouse–Geisser correction was applied to adjust degrees of freedom. For significant ANOVA results, post hoc pairwise comparisons with Bonferroni adjustments were conducted to identify specific differences between time points. Effect sizes were reported using partial eta squared (η^2^) to assess the magnitude of observed effects. Statistical significance was set at *p* < 0.05 for all analyses. All statistical analyses were conducted using the JASP 0.16.4.0 program, and descriptive statistics (means and standard deviations) were calculated for each variable at all time points (see Table 1).

## 3. Results

### 3.1. Descriptive Analysis

The data analysis revealed fluctuations in burnout levels across the three phases. In Phase 1, no elevated levels of burnout were observed, with only 9 out of 37 individuals experiencing moderate levels of burnout. Phase 2, which corresponded to the peak of the COVID-19 pandemic, showed a significant increase in burnout levels, with 18 forensic personnel reporting moderate burnout and 2 individuals exhibiting high burnout levels. In Phase 3, after the pandemic, total burnout levels decreased slightly compared to Phase 2, though they did not return to the low levels observed in Phase 1. During this phase, 1 individual reported high burnout levels, and 11 medical personnel experienced moderate burnout.

A similar pattern was observed across all three burnout subscales, as seen in the total burnout scores. For the EE subscale, Phase 1 results indicated that two individuals experienced high levels of burnout, while nine reported moderate levels. During Phase 2, which coincided with the COVID-19 pandemic, there was a significant increase in EE burnout levels, with 7 medical personnel presenting high levels of burnout and 15 reporting moderate levels. In Phase 3, burnout levels on the EE subscale decreased compared to Phase 2, but they did not return to the low levels observed before the pandemic. In this phase, 2 individuals reported high levels of burnout, and 14 reported moderate levels.

DP levels followed a similar pattern across the three phases of the study. In Phase 1, only 1 individual exhibited high levels of DP burnout, while 10 individuals reported moderate levels on this subscale. Phase 2 showed an increase in DP levels, with five forensic professionals presenting high DP and nine reporting moderate DP levels. In Phase 3, a slight decrease in DP levels was observed compared to Phase 2, with 3 individuals still experiencing high DP and 10 reporting moderate DP levels.

PA scores were lowest during Phase 1, with no cases of high levels of low PA observed. However, 13 individuals reported moderate levels of low PA during this phase. In Phase 2, coinciding with the height of the COVID-19 pandemic, there was an increase in low PA scores, with 20 individuals experiencing moderate levels and 1 individual presenting high levels. By Phase 3, PA scores showed improvement, nearly returning to Phase 1 levels, with 11 individuals exhibiting moderate levels of low PA. High levels of low PA remained rare, with only one case identified in Phase 3.

**Figure 1:**
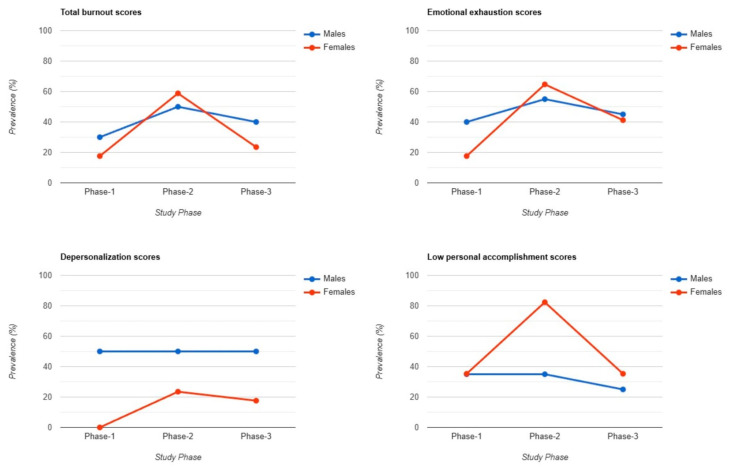
Burnout differences between male and female workers.

Gender comparisons revealed distinct differences in burnout levels across all three phases. In Phase 1, 30% of male forensic workers and 18% of female workers experienced moderate or high total burnout scores. In Phase 2, during the peak of the COVID-19 pandemic, this trend reversed, with more females (59%) experiencing moderate or high burnout levels compared to males (50%). By Phase 3, burnout levels aligned more closely with those observed in Phase 1, with 40% of males and 24% of females reporting moderate or high burnout. The EE subscale followed a similar pattern to total burnout scores. In Phase 1, 40% of males and 18% of females experienced EE, while in Phase 3, these values were 45% for males and 41% for females, showing closely aligned results. However, in Phase 2, emotional exhaustion was more prevalent among females (65%) compared to males (55%) during Phase 2. For DP, 50% of males consistently reported moderate or high levels across all three phases. In contrast, female workers exhibited significant variation: none reported DP in Phase 1, 23% experienced DP in Phase 2, and only 18% in Phase 3. Regarding low PA, females had consistently higher prevalence rates across all three phases. In Phase 2, there was a notable peak, with 82% of females and 35% of males reporting low PA. In Phase 1, levels were equally distributed between genders, with 35% of males and 35% of females experiencing low PA. By Phase 3, low PA levels remained higher in females (35%) compared to males (25%) (Figure 1).

**Figure 2:**
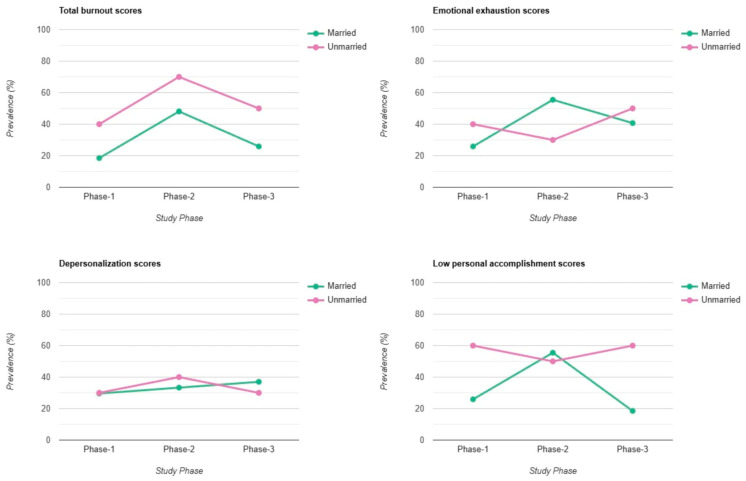
Burnout differences between married and unmarried workers.

Unmarried individuals consistently exhibited higher total burnout scores compared to their married counterparts across all three phases (Phase 1: 40% vs. 18%; Phase 2: 70% vs. 48%; Phase 3: 50% vs. 26%). A similar trend was observed in the Personal Accomplishment (PA) subscale, with unmarried individuals showing lower PA in Phase 1 (60% vs. 26%) and Phase 3 (60% vs. 18%). However, Phase 2 results were more balanced, with married individuals reporting slightly higher levels of low PA (55%) compared to unmarried individuals (50%). The same pattern was reflected in the Emotional Exhaustion (EE) subscale, where married individuals reported higher scores during Phase 2 (55%) compared to unmarried individuals (30%). In contrast, during Phase 1 and Phase 3, EE scores were higher among unmarried workers. For Depersonalization (DP), medium and high scores were more evenly distributed between married and unmarried forensic workers across all phases (Phase 1: 30% vs. 30%; Phase 2: 40% vs. 33%; Phase 3: 30% vs. 37%) (Figure 2).

**Figure 3:**
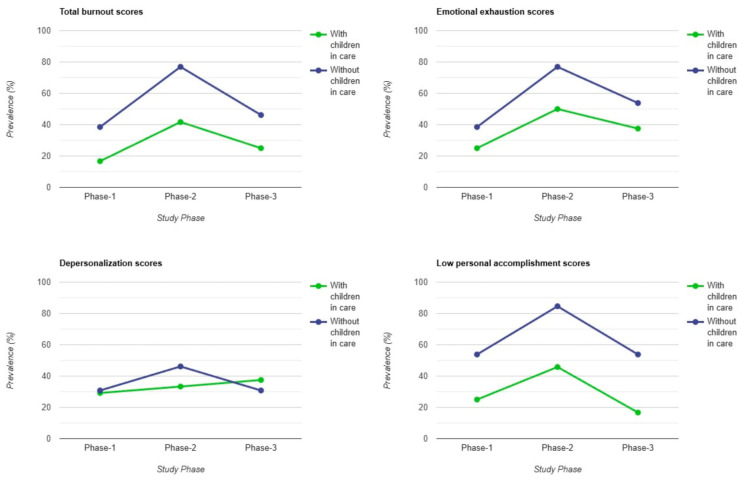
Burnout differences between workers with and without children in care.

Additionally, forensic workers with children in their care exhibited lower burnout prevalence in total scores and all subscales, except DP levels, where results were more evenly distributed between workers with and without children (Figure 3).

**Figure 4:**
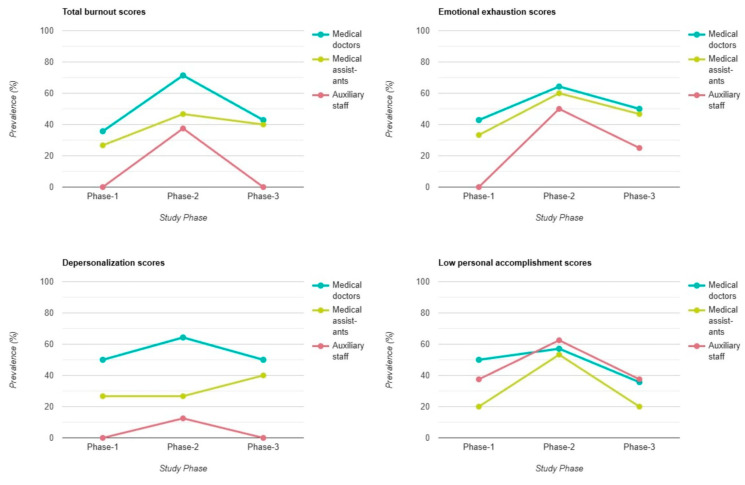
Burnout differences between different professional groups.

No distinctive patterns were observed in total burnout scores or in the results of the three subscales when comparing different age groups. However, when examining professional groups, the findings revealed that medical doctors consistently experienced moderate or high levels of total burnout across all three phases, with a peak of 71% in Phase 2 during the pandemic. Medical assistants followed while auxiliary staff reported the lowest levels of burnout. A similar pattern was evident in the EE and DP subscales. The only deviation from this trend occurred in the PA subscale. In this case, auxiliary staff reported lower PA levels than medical doctors in Phase 2 (62% vs. 57%) and Phase 3 (37% vs. 36%) (Figure 4).

Four supervisors participated in the study, including two medical doctors and two medical assistants. In Phase 1, 3 out of 4 supervisors exhibited moderate levels of total burnout. During Phase 2, at the pandemic’s peak, three supervisors continued to show moderate burnout levels, while one presented high levels of total burnout. By Phase 3, the pattern remained consistent, with three supervisors exhibiting moderate burnout. Notably, one supervisor displayed high DP across all three phases, while high EE levels were observed in two supervisors during Phase 2.

### 3.2. Statistical Analysis

The analysis revealed a significant effect of time on emotional exhaustion levels, F(1.16, 41.93) = 15.96, *p* < 0.001, η^2^ = 0.307, indicating that scores differed significantly across the three time points. Post hoc pairwise comparisons using the Bonferroni correction showed a statistically significant difference between Phase 1 and Phase 2, with a mean difference of −4.757 (95% CI: −6.825, −2.688), t(36) = −5.637, *p* < 0.001, between Phase 1 and Phase 3, with a mean difference of −2.081 (95% CI: −4.150, −0.013), t(36) = −2.466, *p* = 0.048, and between Phase 2 and Phase 3, with a mean difference of 2.676 (95% CI: 0.607, 4.744), t(36) = 3.172, *p* = 0.014 (Table 2).

For depersonalization, the results were not significant, F(1.10, 39.84) = 3.21, *p* = 0.077, suggesting no meaningful changes in scores across the three time points.

A significant main effect of time was observed for personal achievement, F(1.23, 44.31) = 9.686, *p* = 0.002, η^2^ = 0.212. Pairwise comparisons indicated a statistically significant difference between Phase 1 and Phase 2, with a mean difference of −2.757 (95% CI: −4.449, −1.064), t(36) = −5.992, *p* < 0.001, and between Phase 2 and Phase 3, with a mean difference of 2.486 (95% CI: 0.794, 4.179), t(36) = 4.179, *p* = 0.003. However, the difference between Phase 1 and Phase 3 was not statistically significant, with a mean difference of −0.270 (95% CI: −1.963, 1.422), t(36) = −0.391, *p* = 1.000 (Table 3).

The analysis revealed a significant effect of time on total burnout score, F(1.12, 40.58) = 23.81, *p* < 0.001, η^2^ = 0.398, indicating that scores differed significantly across the three time points. Post hoc pairwise comparisons using the Bonferroni correction revealed a statistically significant difference between Phase 1 and Phase 2, with a mean difference of −9.081 (95% CI: −12.368, −5.794), t(36) = −6.772, *p* < 0.001, and between Phase 2 and Phase 3, with a mean difference of 6.081 (95% CI: 2.794, 9.368), t(36) = 4.535, *p* < 0.001. However, the difference between Phase 1 and Phase 3 was not statistically significant, with a mean difference of −3.000 (95% CI: −6.287, 0.287), t(36) = −2.237, *p* = 0.085 (Table 3).

## 4. Discussion

The findings of our study confirm our initial hypotheses, indicating that burnout peaked during the middle phase of the pandemic, with significant increases in emotional exhaustion, followed by a partial decrease post-pandemic. Gender-based differences were observed, with female professionals reporting higher emotional exhaustion while male professionals exhibited higher depersonalization levels. Also, the results suggest that married individuals and those with children experienced lower burnout levels during the pandemic. Medical doctors reported the highest burnout levels, particularly in terms of emotional exhaustion.

In Phase 1 (September 2020), no participants exhibited high total burnout scores, and 24% reported moderate levels of burnout. These findings are similar to results from other studies from and before 2020. Iorga et al. studied burnout syndrome in forensic medicine in 2016 [20] and 2020 [21], with results suggesting that forensic workers had low burnout risk in Romania at that time. Also, close to 20% of emergency management, law enforcement, and criminal justice personnel, who also work in the same scenarios as forensic doctors and pathologists, develop or are at high risk of occupational stress, which can generate burnout [22]. During Phase 2, at the height of the pandemic, total burnout levels peaked, with 49% experiencing moderate burnout and 5% reporting high burnout levels. Studies during the pandemic concluded that the overall burnout score within the pathology department was 58%, a value close to our own [23]. Another review conducted by Khatab et al. found similar patterns of burnout prevalence in the pathology department during that time period [12]. An explanation can be that the combination of increased workload, psychological distress, and lack of institutional support created ideal conditions for burnout among forensic pathologists, with acute stressors such as infection risk, ethical dilemmas, and high case volume likely contributing to these results [24].

The results revealed significant changes in emotional exhaustion, personal achievement, and total burnout scores over time but not in depersonalization. Significant fluctuations were observed in total burnout levels across the three phases, with a peak observed during Phase 2, coinciding with the height of the COVID-19 pandemic. Statistically, a significant effect of time was found, with significant differences observed between Phase 1 and Phase 2 and between Phase 2 and Phase 3, indicating a notable change in burnout levels over time. Regarding emotional exhaustion, there was a significant effect of time, with post hoc comparisons showing significant differences between Phase 1 (September 2020) and Phase 2 (October 2021), as well as between Phase 1 and Phase 3 (October 2024). The difference between Phase 2 and Phase 3 was also significant, indicating emotional exhaustion fluctuations across the studied period. In other words, total burnout scores and EE levels increased substantially during Phase 2, while a partial recovery was noted in Phase 3, though levels did not return to the baseline observed in Phase 1. DP levels followed a similar trend, with moderate increases during Phase 2 and slight declines in Phase 3, but with no significant statistical effect of time on the scores, suggesting that depersonalization levels did not change meaningfully across the three time points. A potential explanation for this lack of statistical significance is self-selection bias. However, in this study, self-selection bias is unlikely to be a major concern, as 37 out of 43 forensic and pathology personnel (86%) in Sibiu County completed the questionnaire. This high response rate suggests that the sample is highly representative of the local workforce, minimizing concerns about systematic underreporting or selective participation. In contrast, some expected effects may not have been significant due to workplace dynamics, where employees with strong peer support and leadership engagement may have been better equipped to manage stress. Additionally, the presence of effective coping mechanisms or greater resilience among employees could have contributed to stabilizing depersonalization levels during the COVID-19 crisis, particularly among men, where depersonalization scores were high but remained constant. Studies show that mindfulness techniques, social support, and active coping strategies are among the most effective protective factors against burnout, and professionals who engaged in problem-solving approaches, professional collaboration, and peer support networks reported lower levels of depersonalization. Additionally, humor, emotional support from colleagues, and positive reframing were found to lower the psychological impact of exposure to traumatic cases [11]. Future studies should further investigate this hypothesis. A significant main effect was also observed in PA scores, with significant differences between Phase 1 and Phase 2, as well as between Phase 2 and Phase 3. However, no significant difference was found between Phase 1 and Phase 3, suggesting changes in personal achievement primarily occurred between the first and second time points. Low PA was most prevalent in Phase 2, particularly among females and auxiliary staff.

Regarding power analysis, the reported η^2^ values suggest medium to large effect sizes for the significant findings, indicating that the study was adequately powered to detect these effects. The significant effects across multiple variables and the post hoc results strengthen the conclusion that time had a meaningful impact on burnout and related subscales, except for depersonalization. For the total burnout score, emotional exhaustion, and personal achievement, the analyses showed significant effects of time, with medium to large effect sizes (η^2^ = 0.398, η^2^ = 0.307, and η^2^ = 0.212, respectively). These significant effects indicate that the changes in burnout levels, emotional exhaustion, and personal achievement across the three time points in the study provide substantial evidence that time is a key factor influencing these aspects of burnout. The η^2^ values further substantiate the practical relevance of these effects, with personal achievement showing a medium effect size and total burnout levels and emotional exhaustion showing larger effect sizes. In contrast, the effect for depersonalization was not significant (*p* = 0.077), indicating that there were no meaningful changes in depersonalization scores over time. This finding suggests that depersonalization may not have been as sensitive to the time points assessed, or other factors may be more influential in this dimension of burnout. Future research could further explore these aspects and consider additional factors influencing depersonalization in burnout studies.

In summary, the repeated measures ANOVA revealed that emotional exhaustion, personal achievement, and total burnout scores changed significantly over time, with substantial effect sizes. Specifically, emotional exhaustion showed a significant time effect with a large effect size (η^2^ = 0.307), indicating that participants’ levels of emotional exhaustion varied markedly across the three phases. Similarly, personal achievement and total burnout demonstrated significant time effects with moderate to large effect sizes (η^2^ = 0.212 and η^2^ = 0.398, respectively). These effect sizes suggest that the observed changes in these burnout dimensions are not only statistically significant but also practically meaningful.

In contrast, depersonalization did not exhibit a significant change over time (*p* = 0.077), implying that this particular dimension remained relatively stable across the study period. This pattern of findings suggests that while certain aspects of burnout (emotional exhaustion, personal achievement, and overall burnout) are sensitive to temporal changes, depersonalization may be less responsive under the conditions examined.

We conducted a post hoc power analysis using G*Power, setting the alpha level at 0.05 and an expected effect size of f = 0.25. With a sample size of 37 participants, the analysis yielded an achieved power of approximately 66.54%. This result indicates that assuming the expected effect size is accurate, there is a 66.54% probability of correctly rejecting the null hypothesis when a true effect exists. However, since the conventional threshold for adequate power is typically set at 80%, our study’s power suggests an increased risk of committing a Type II error—that is, failing to detect a true effect. These findings imply that while our study provides valuable insights, it may be underpowered to detect smaller but potentially meaningful effects. Consequently, non-significant findings should be interpreted with caution, and future research with a larger sample size is recommended to enhance the robustness of the results.

As with our work, several studies have highlighted the significant impact of the COVID-19 pandemic on burnout levels across various healthcare professionals. Bogaerts et al. found that healthcare workers faced heightened emotional job demands and distress during the pandemic [25]. They noted that individuals with high resilience levels experienced less burnout, suggesting the importance of individual psychological factors in coping with pandemic-related stress [25]. Ghahramani et al. noted a pooled burnout prevalence of 52% [26]. A similar pattern was observed by Gualano et al., where burnout rates ranged from 49.3% to 58% among ICU/ED professionals [27]. Zhang et al. (2020) reported a burnout prevalence of 57.5% among frontline workers [28], particularly those directly interacting with COVID-19 patients [29]. In comparison to our results, burnout levels in forensic medicine and pathology during the pandemic seem to be similar to those of first-line healthcare workers. EE and DP were the most prominent dimensions in healthcare workers during the pandemic [26,27,29,30] in contrast to our results, where EE and PA levels were most prominent. These studies identified a combination of high workload, fear of infection, and lack of support as key contributors to burnout [27]. Also, burnout levels were higher during the COVID-19 pandemic compared to previous outbreaks like SARS and MERS [31].

Gender differences were prominent, with slightly higher levels of burnout in male workers during the first Phase but with females experiencing much higher burnout levels during Phase 2, especially on the EE subscale, while males showed consistently higher DP levels across all phases. Studies examining gender differences in burnout across healthcare settings have consistently found the same pattern. Carmassi et al. reported that female physicians are at greater risk of burnout, particularly in emotional exhaustion [32]. Similarly, Doraiswamy et al. observed that female physicians experienced more emotional exhaustion, which correlated with higher overall burnout scores [33]. Other articles found that female healthcare workers reported significantly higher emotional exhaustion and depersonalization, while no gender differences were noted in personal accomplishment [34]. In contrast, male physicians reported higher levels of depersonalization [35], which is similar to our results. Other studies have found no significant differences in overall burnout levels between males and females [36].

Unmarried individuals consistently exhibited higher burnout scores than their married counterparts, except for EE scores, where married individuals surpassed their unmarried counterparts in Phase 2. Cañadas-De la Fuente found that single or divorced individuals exhibited higher levels of burnout, particularly in emotional exhaustion and depersonalization, compared to married individuals [37]. This aligns with the findings of Zarei et al., who reported that single employees were more than three times as likely to experience high burnout levels than married employees, particularly in emotional exhaustion and depersonalization [38]. In contrast, Chen et al. observed that while marital status had a moderate effect on personal burnout, married individuals reported slightly higher burnout levels, though the difference was not statistically significant [39]. Similarly, another article found that marital status played a lesser role in predicting burnout, with only a slight trend indicating that married individuals experienced lower burnout levels compared to their single counterparts [40].

Forensic and pathology workers with children in their care demonstrated lower overall burnout prevalence, and among professional groups, medical doctors reported the highest burnout levels, with a notable peak in Phase 2, followed by medical assistants and auxiliary staff. On the PA subscale, the results indicated that auxiliary staff scores surpass those of medical doctors and medical assistants in Phase 2 and Phase 3. Other studies from the literature found that nurses consistently reported higher burnout levels compared to doctors. Specifically, nurses were more likely to experience emotional exhaustion and depersonalization, while doctors were more likely to report reduced personal accomplishment [33,37,40]. In contrast, Olanrewaju and Chineye reported that medical doctors in their study experienced significantly higher levels of emotional exhaustion and depersonalization than medical assistants. However, the burnout levels of medical assistants were still high in terms of emotional exhaustion [34]. Similar results were reported by other studies [35,36].

Supervisors displayed persistent moderate burnout levels across all three phases, with some reporting high EE and DP during Phase 2. Results from the literature are in contrast with our findings. Supervisors in medical settings reported significantly lower EE and DP levels than staff members [41]. Also, head nurses exhibited significantly lower EE and DP scores than senior nurses, with high emotional exhaustion observed in 28% of head nurses compared to 59% of senior nurses [42]. Wu et al. further corroborated these findings, showing that head doctors had significantly lower EE and cynicism scores than staff doctors [43]. These results may be attributed to head doctors’ and nurses’ greater autonomy, job control, and decision-making authority. However, one explanation for the differing results in our study could be the impact of the COVID-19 pandemic, as most studies in the literature on this topic were conducted during pre-pandemic years. The crisis brought on by the pandemic placed significant pressure on head doctors and nurses, particularly in the form of crisis decision-making.

The study has several limitations that we need to take into consideration. First, the small sample size may limit the generalizability of the findings, as it reduces the ability to capture a broader range of experiences and burnout patterns across forensic and pathology personnel. Second, being a single-center study, the results may not reflect the conditions or challenges faced by similar professionals in other regions or institutions with differing organizational structures, resource availability, or pandemic responses. The lack of pre-pandemic baseline data limits the ability to comprehensively assess how burnout levels evolved over time, as Phase 1 of the study represents a period shortly after the initial wave of the COVID-19 pandemic rather than an actual pre-pandemic reference point. Also, we need to mention the potential for self-reporting bias, as burnout levels were assessed using self-reported questionnaires. Responses may have been influenced by individual perception, recall bias, or mood, where participants might have underreported or exaggerated their symptoms based on personal or professional concerns. Lastly, the absence of long-term longitudinal data limits our ability to assess whether burnout symptoms persisted, worsened, or resolved over time. A longer follow-up period would provide a more comprehensive understanding of recovery trajectories and the effectiveness of workplace adaptations.

While this study provides valuable insights into burnout among forensic medicine and pathology personnel in a single-center setting across multiple time points during the COVID-19 pandemic, future research should aim to expand the scope of investigation by including multiple forensic and pathology institutions across the country but also across the world. A broader, multi-center study would allow for comparisons between different institutional environments, examining how factors such as organizational structure, workload distribution, and regional variations in forensic case exposure influence burnout levels. Additionally, further research should explore longitudinal trends to assess whether burnout levels fluctuate over extended periods and how various intervention strategies impact psychological resilience in forensic and pathology professionals.

## 5. Conclusions

- The COVID-19 pandemic significantly influenced burnout levels among forensic medicine and pathology personnel, with the highest scores recorded during its peak (Phase 2);
- Burnout levels decreased post-pandemic; they did not return to pre-pandemic levels, indicating a lasting psychological impact;
- Emotional exhaustion was the most affected, rising sharply during the crisis and showing only partial recovery afterward;
- Personal accomplishment also declined, particularly in the early to middle stages of the pandemic, while depersonalization remained stable across all phases;
- Females experienced higher emotional exhaustion during the peak of the pandemic, while males exhibited consistently higher depersonalization levels;
- Unmarried individuals and those without children were more vulnerable to burnout, highlighting the protective role of familial support;
- Medical doctors reported the highest burnout levels, largely due to their leadership responsibilities and crisis decision-making roles, while auxiliary staff struggled with maintaining personal accomplishment.

The prolonged and intense nature of the COVID-19 pandemic likely exacerbated the chronic stress and burnout experienced by forensic and pathology staff. Stressors associated with the pandemic, including changes in work, increased workload, high death tolls, crisis decision-making, social isolation, and uncertainty, had a substantial impact on mental health and well-being during as well as after the pandemic’s peak.

The findings of this study have direct implications for forensic medicine and pathology professionals, as burnout can negatively impact job performance, decision-making, and overall well-being. Increased emotional exhaustion and depersonalization may lead to decreased efficiency, lower accuracy in forensic evaluations, and ethical concerns in handling sensitive cases. Given the high workload and exposure to traumatic cases, forensic professionals require institutional support systems to maintain professional effectiveness and prevent long-term psychological distress.

The observed fluctuations in burnout levels highlight the need for targeted mental health interventions, particularly during peak stress periods such as pandemics. The gender-based differences in burnout symptoms suggest gender-based strategies regarding workplace well-being. The protective effect of family status on burnout levels emphasizes the importance of work-life balance policies, including flexible scheduling and institutional support for employees with children. Furthermore, it seems that psychological distress persists beyond the immediate crisis; thus, workers need long-term monitoring and support programs. Overall, there is an acute need for organizational reforms, such as improved workload distribution, peer support networks, and mental health training, to build resilience among forensic and pathology personnel. Future research should explore longitudinal recovery patterns and assess the effectiveness of specific interventions in reducing occupational burnout in crisis settings.

## Figures and Tables

**Table 1:** Demographic characteristics.

Characteristic	Male	Female	Urban	Rural	Single	In a Relationship	Married	Divorced	Widowed	Children
N	20	17	34	3	0	4	27	5	1	24
Mean	0.541	0.459	0.919	0.081	0	0.108	0.73	0.135	0.027	0.649
%	54.05%	45.90%	91.90%	8.10%	0%	10.80%	73%	13.50%	2.70%	64.90%

**Table 2:** The levels of burnout by dimensions.

Dimension	Low Level	Medium Level	High Level
Emotional exhaustion	9–18	19–27	28–45
Depersonalization	6–12	13–18	19–30
Reduced personal accomplishment	10–20	21–30	31–50
Total score	25–50	51–75	76–125

**Table 3:** Descriptive statistics.

	Emotional Exhaustion Phase 1	Emotional Exhaustion Phase 2	Emotional Exhaustion Phase 3	Depersonalization Phase 1	Depersonalization Phase 2	Depersonalization Phase 3	Personal Achievement Phase 1	Personal Achievement Phase 2	Personal Achievement Phase 3	Total Phase 1	Total Phase 2	Total Phase 3
N	37	37	37	37	37	37	37	37	37	37	37	37
Mean	16.32	21.08	18.40	10.67	12.24	11.32	18.54	21.29	18.81	45.54	54.62	48.54
Std. Deviation	5.49	6.68	5.28	4.21	4.54	4.39	4.13	4.83	4.40	10.14	12.15	10.39
Minimum	9.00	10.00	11.00	6.00	6.00	6.00	11.00	13.00	11.00	27.00	38.00	30.00
Maximum	31.00	41.00	32.00	19.00	24.00	20.00	29.00	32.00	31.00	74.00	94.00	79.00
95% CI (Lower–Upper)	14.50–18.14	18.88–23.28	16.63–20.17	9.21–12.13	10.66–13.82	9.79–12.85	17.16–19.92	19.66–22.92	17.28–20.34	41.99–49.09	50.33–58.91	44.89–52.19

## Data Availability

The raw data supporting the conclusions of this article will be made available by the authors upon request.

## Abbreviations

The following abbreviations are used in this manuscript:

EE	Emotional Exhaustion
DP	Depersonalization
PA	Personal Accomplishment
PTSD	Post-traumatic Stress Syndrome
FMW	Forensic Medicine Workers
